# Association of 25-Hydroxyvitamin D With Metabolic Dysfunction-Associated Fatty Liver Disease: Results From NHANES 2017–2018

**DOI:** 10.1155/ije/1368301

**Published:** 2025-09-23

**Authors:** Xiaojuan Rao, Xinxin Zhang, Shuo Li, Bo Huang, Junhe Wang, Jingqiu Cui, Ming Liu, Tiekun Yan

**Affiliations:** ^1^Department of Endocrinology and Metabolism, Tianjin Medical University General Hospital, Tianjin, China; ^2^Department of Nephrology, Kidney Disease Medical Center, National Key Clinical Specialty, Tianjin Key Medical Discipline, Tianjin Medical University General Hospital, Tianjin, China

**Keywords:** 25-Hydroxyvitamin D, 25-Hydroxyvitamin D_3_, metabolic dysfunction-associated fatty liver disease, National Health and Nutrition Examination Survey

## Abstract

**Background:** The association of vitamin D with metabolic dysfunction-associated fatty liver disease (MAFLD) remained unclear. This study aimed to examine the relationships of total 25-hydroxyvitamin D [25(OH)D, the sum of 25(OH)D_2_ and 25(OH)D_3_], 25(OH)D_3_, and epi-25(OH)D_3_ with MAFLD.

**Methods:** We used the National Health and Nutrition Examination Survey of the 2017–2018 cycle for our present analysis. Binary logistic regression analyses were conducted to explore the associations of total 25(OH)D, 25(OH)D_3_, and epi-25(OH)D_3_ with MAFLD after adjusting for confounders. Interaction tests were conducted to compare the association between 25(OH)D and MAFLD in subgroups.

**Results:** The final analysis included 4605 subjects. After adjustment for confounders, the odds of MAFLD decreased with increasing concentrations of total 25(OH)D (odds ratio [OR], 0.45; 95% confidence interval [CI], 0.29–0.68; *p* for trend < 0.001). Serum 25(OH)D_3_ showed a strong inverse association with MAFLD (OR, 0.39; 95% CI, 0.25–0.59; *p* for trend < 0.001). In addition, participants with lower epi-25(OH)D_3_ levels had a higher likelihood of MAFLD, as demonstrated in both quartile and continuous models (OR, 0.77; 95% CI, 0.60–0.99; *p*=0.041). After stratification by lipid status, inverse associations of total 25(OH)D and 25(OH)D_3_ with MAFLD were found only in dyslipidemic participants (*p* for interaction < 0.001). A sex-specific interaction was also noted for 25(OH)D_3_, showing stronger effects in women than in men (*p*=0.036).

**Conclusions:** Low serum total 25(OH)D, 25(OH)D_3_, and epi-25(OH)D_3_ were significantly associated with a higher prevalence of MAFLD. These associations showed nonlinear patterns and were particularly evident among participants with dyslipidemia, with 25(OH)D_3_ demonstrating stronger protective effects in women than in men. Given the cross-sectional design, causality cannot be inferred, and further prospective studies are required to confirm these findings.

## 1. Introduction

Metabolic dysfunction-associated fatty liver disease (MAFLD) refers to fatty liver disease linked with systemic metabolic disorders, which is a new concept suggested in 2020 [1]. MAFLD is not only a simple rename of nonalcoholic fatty liver disease (NAFLD), which is distinct in that it encompasses metabolic dysfunction and that it is unrelated to alcohol use and co-occurring causes of liver disease. The change in definition will help identify metabolically complex fatty liver with concomitant chronic liver disease or alcohol use and rule out fatty liver not related to metabolic disorders. Although the change in concept may seem subtle, the resulting effect can have major implications on our current understanding of prevalence and risk factors [2].

Vitamin D, a kind of steroid hormone, regulates the balance of calcium and phosphorus, decreases insulin resistance and the odds of obesity and diabetes mellitus (DM), and suppresses oxidative stress, inflammation, and autoimmune disorders [3–5]. Circulating 25-hydroxyvitamin D [25(OH)D] is regarded as a clinical indication of vitamin D status [6]. 25(OH)D mainly includes 25(OH)D_2_ and 25(OH)D_3_. 25(OH)D_2_ and 25(OH)D_3_ are the most important vitamin D compounds in the human body. They are obtained from diet and supplements or synthesized in the epidermis of the skin. Epi-25(OH)D_3_ is an isomer of 25(OH)D_3_ with a 3α- instead of a 3β-hydroxyl, which affects the bioactive conversion rate of vitamin D [7]. Recent studies have shown that changes in epi-25(OH)D_3_ levels may regulate cardiovascular functional capacity in patients with advanced chronic kidney disease [8]. Earlier investigations mainly concentrated on the associations of total 25(OH)D with NAFLD and severe liver fibrosis. According to observational studies, low levels of 25(OH)D have been shown to link to a greater risk of developing NAFLD and severe liver fibrosis, while appropriate levels will speed up the resolution of NAFLD [9, 10]. However, prior research did not distinguish between different metabolites of total 25(OH)D, leaving the associations of total 25(OH)D and its metabolites with MAFLD unclear.

Thus, the purpose of this study was to investigate the associations of total 25(OH)D [which is the sum of 25(OH)D_2_ and 25(OH)D_3_], 25(OH)D_3_, and epi-25(OH)D_3_ with MAFLD based on the National Health and Nutrition Examination Survey (NHANES) 2017–2018.

## 2. Materials and Methods

### 2.1. Study Population

The NHANES is a nationally representative study that evaluates the nutrition and physical health of the non-institutionalized U.S. population. Briefly, NHANES was conducted every 2 years by the National Center for Health Statistics of the Centers for Disease Control and Prevention (https://wwwn.cdc.gov/nchs/nhanes/analyticguidelines.aspx). Herein, we used data from 2017 to 2018 NHANES cycle for our present analysis because the infromation of liver ultrasound transient elastography and 25(OH)D was specifically included. The study procedure was approved by the Ethics Review Board of the National Center for Health Statistics, and every participant signed informed consent.

Participants included in the study were aged 20 years or older and had completed data on 25(OHD) and ultrasound data controlled attenuated parameter (CAP). The exclusion criteria were: (1) aged less than 20 years; (2) incomplete data on the CAP; (3) no data on total 25(OH)D, 25(OH)D_3_ or epi-25(OH)D_3_; and (4) history of liver cancer or viral hepatitis.

### 2.2. Data Extraction

Data obtained from the questionnaire included age, sex, ethnicity, education level and household income–poverty ratio, smoking and drinking status, and physical activity. Body weight, height, waist circumference, and blood pressure were obtained from physical examinations. BMI ≥ 30 kg/m^2^ was used to characterize obesity. Levels of lipid profiles, fasting plasma glucose (FPG), fasting serum insulin, glycosylated hemoglobin (HbA1c), liver enzymes (alanine aminotransferase [ALT] and aspartate aminotransferase [AST]) and hypersensitive C reactive protein (hs-CRP) were gathered from laboratory data. The formula used to determine HOMA-IR was (fasting serum insulin [mU/L] × FPG [mmol/L])/22.5 [11].

Physical activity was assessed by the frequency of participants' engagement in physical activities per week and the corresponding metabolic equivalents (METs). The participants were separated into 4 groups based on the value of METs (MET min/week): low (< 600), moderate (600–8000), high (≥ 8000), and those with missing values [12].

### 2.3. Exposure

The quantities of total 25(OH)D, 25(OH)D_2_, 25(OH)D_3_, and epi-25(OH)D_3_ were determined using high-performance liquid chromatography-tandem mass spectrometry. The sum of 25(OH)D_2_ and 25(OH)D_3_ is known as total 25(OH), however, epi-25(OH)D_3_ is not included. Participants were categorized into four groups based on their serum total 25(OH)D levels: vitamin D deficiency group (< 30 nmol/L), vitamin D inadequate group (≥ 30, < 50 nmol/L), vitamin D adequate group (≥ 50, ≤ 120 nmol/L), and vitamin D excess group (> 120 nmol/L) [13]. The limit of detection of 25(OH)D_2_ was 1.45 nmol/L. There were 3668 of 4605 individuals with levels of 25(OH)D_2_ below 1.45 nmol/L. Thus, we divided the population into two groups by 25(OH)D_2_ levels: ≤ 1.45 and > 1.45 nmol/L. In addition, data on the time of sample collection were extracted and categorized into two periods: May 1 through October 31 and November 1 through April 30.

### 2.4. Definitions

DM was defined as FPG ≥ 7.0 mmol/L, 2 h plasma glucose (2h-PG) ≥ 11.1 mmol/L, HbA1c ≥ 6.5%, self-reported history of DM, or the use of hypoglycemic medications [14]. Prediabetes was defined as an FPG level between 5.6 and 6.9 mmol/L, a 2h-PG between 7.8 and 11.0 mmol/L, or an HbA1c level between 5.7% and 6.4% [14]. Dyslipidemia was diagnosed when meeting one or more of the following criteria: total cholesterol (TC) level ≥ 5.18 mmol/L, triglyceride (TG) level ≥ 1.7 mmol/L, low-density lipoprotein cholesterol (LDL-C) level ≥ 3.37 mmol/L, high-density lipoprotein cholesterol (HDL-C) level < 1.04 mmol/L for males and < 1.30 mmol/L for females, or the use of specific drug treatment.

### 2.5. Outcome

Based on the hypothesis that fat affects the propagation of ultrasound, the controlled attenuation parameter (CAP) is a physical measurement derived from the ultrasound signal collected using FibroScan. CAP is used to assess liver steatosis [15]. In NHANES 2017–2018, hepatic steatosis-related CAP measurements were conducted using the FibroScan 502 Touch device. The diagnostic cutoff value for CAP to detect the presence of hepatic steatosis was set at 248 dB/m [16]. MAFLD was defined as the presence of hepatic steatosis combined with at least one of the following criteria [1]: (i) being overweight or obesity [body mass index (BMI) ≥ 25 kg/m^2^ in Caucasians or ≥ 23 kg/m^2^ in Asians]; (ii) having diabetes; and (iii) having ≥ 2 risk factors of metabolic abnormalities: (a) waist circumference ≥ 102/88 cm in Caucasian men and women (or ≥ 90/80 cm in Asian men and women); (b) hypertension (≥ 130/85 mmHg or receiving specific drugs); c) TG ≥ 1.70 mmol/L or with specific drugs; (d) HDL-C level (< 1.0 mmol/L in men and< 1.3 mmol/L in women); (e) prediabetes; (f) homeostasis model assessment of insulin resistance (HOMA-IR) score ≥ 2.5; and (g) hs-CRP > 2 mg/L.

### 2.6. Statistical Analysis

The 2-year sample weights were used to represent the stratified, multistage probability design of the NHANES. Categorical variables were displayed as numbers and percentages, whereas continuous ones were given as means ± standard error (SE). Two-sample *t*-tests and chi-square tests were employed to compare groups for continuous and categorical variables, respectively. Binary logistic regression analyses were conducted to explore the associations of total 25(OH)D, 25(OH)D_3,_ and epi-25(OH)D_3_ with MAFLD in two models (Model 1: unadjusted; Model 2: adjusted for age and gender; Model 3: adjusted for age, gender, race, education levels, family income–poverty ratio, physical activity levels, smoking and drinking status, and the sampling season). Total 25(OH)D, 25(OH)D3, and epi-25(OH)D3 are included in 3 models as continuous variables (log-transformed with base natural constant) and categorical variables (quartiles, with the first quartile as the reference), respectively.

A restricted cubic spline with four knots was employed to model the effect of 25(OH)D on MAFLD and assess possible nonlinearity associations. The levels of vitamin D vary with age, gender, and BMI [17]; vitamin D is associated with chronic diseases, including DM and cardiovascular diseases [18]. Therefore, subgroup analyses were carried out within the following categories: age (≤ 45, > 45 y), sex (female, male), obesity (no, yes), DM (no, yes), hypertension (no, yes), and dyslipidemia (no, yes). The relationship between 25(OH)D and MAFLD in different subgroups was compared using interaction tests in the logistic regression analysis. R software (Version 4.2.2; R Foundation, Vienna, Austria) was used for all the analyses. Statistical significance was computed using a two-sided *p* value < 0.05.

## 3. Results

**Figure 1:**
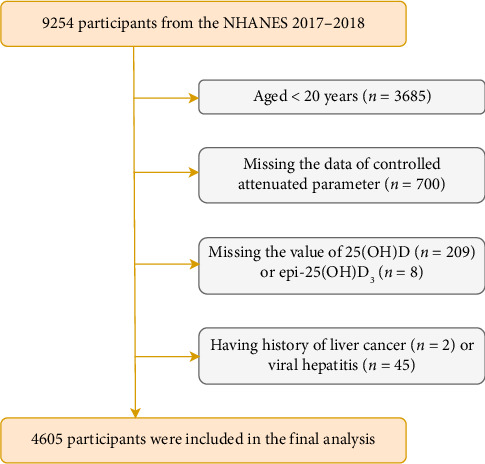
Flow chart of identification of study population. There were 4605 individuals in total included in the present analysis.

In total, this cycle comprised 9254 participants. After excluding participants aged less than 20 years (*n* = 3685), lack of data on the mean CAP (*n* = 700), those without measurement of 25(OH)D (*n* = 209) and epi-25(OH)D_3_ (*n* = 8), and those with a history of liver cancer (*n* = 2) or viral hepatitis (*n* = 45), the present analysis included 4605 individuals in total (Figure 1).

### 3.1. Characteristics of Participants With and Without MAFLD

Table 1 showed characteristics of participants by the status of MAFLD. The weighted prevalence of MAFLD was 54.91%. The study population had a mean age of 48.01 years, with 48.80% being men. Mean serum total 25(OH)D, 25(OH)D_3_, and epi-25(OH)D_3_ levels were 73.06 ± 1.60 nmol/L, 69.48 ± 1.59 nmol/L, and 4.59 ± 0.17 nmol/L, respectively. Individuals with MAFLD were more likely to be older, male, and obese (all *p* < 0.001). They also had lower levels of education and physical activity, along with a higher prevalence of hypertension, diabetes, and dyslipidemia compared with those without MAFLD (all *p* < 0.010). Biochemical parameters further showed that participants with MAFLD had higher ALT levels (26.24 ± 0.62 vs. 19.34 ± 0.60 U/L, *p* < 0.001), while AST levels did not differ significantly between groups (22.88 ± 0.43 vs. 21.30 ± 0.52 U/L, *p*=0.066). In terms of vitamin D metabolites, individuals with MAFLD exhibited lower 25(OH)D_3_ than healthy controls (*p*=0.023), whereas no significant differences were observed for total 25(OH)D and epi-25(OH)D_3_ (all *p* > 0.050).

### 3.2. Associations of Total 25(OH)D, 25(OH)D_3_, and epi-25(OH)D_3_ With MAFLD

Table 2 presents the odds ratios (ORs) and confidence intervals (CIs) for the association of total 25(OH)D, 25(OH)D_3,_ and epi-25(OH)D_3_ with MAFLD. When total 25(OH)D was categorized into quartiles, the odds of MAFLD reduced with an increasing total 25(OH)D concentration after adjustment for age, sex, race, education levels, physical activity, family income–poverty ratio, smoking and drinking conditions, and the sampling season in Model 3 (OR, 0.45 [0.29–0.68]; *p* for trend < 0.001). Consistently, this association remained statistically significant even after natural log-transformation of total 25(OH)D (OR, 0.54 [0.40, 0.73]; *p* < 0.001). Moreover, when compared to individuals in the vitamin D adequate group, those in vitamin D inadequate groups were associated with MAFLD (deficiency vs. adequate: 1.63 [1.19, 2.23]; *p*=0.005, Supporting Table 1).

In Model 3, serum 25(OH)D_3_ levels exhibited a negative association with the prevalence of MAFLD ([OR, 0.39 (0.25, 0.59)]; *p* for trend < 0.001). In addition, the risk of MAFLD was still 48% lower per unit increase in natural log-transformed 25(OH)D_3_ (OR, 0.52 [0.38, 0.72]; *p* < 0.001). After adjusting for covariates, low epi-25(OH)D_3_ levels were associated with the odds of MAFLD (OR, 0.63 [0.42, 0.95]; *p* for trend = 0.029). Moreover, when analyzed as a continuous variable, this link was still significant (OR, 0.77 [0.60, 0.99]; *p*=0.041Table 2).

**Figure 2:**
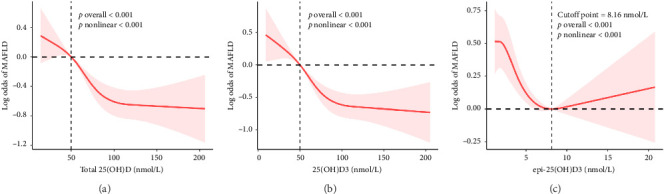
Restricted cubic spline analyses of serum vitamin D metabolites and the odds of MAFLD. Total 25(OH)D and 25(OH)D_3_ showed significant nonlinear inverse associations with MAFLD (both *p*-overall < 0.001, *p*-nonlinear < 0.001), while epi-25(OH)D_3_ demonstrated a nonlinear relationship with a cutoff at 8.16 nmol/L, below which it was inversely associated with MAFLD, whereas no significant association was observed above this level (*p*-overall < 0.001, *p*-nonlinear < 0.001).

The dose-response relationship between 25(OH)D and MAFLD is depicted by the restricted cubic splines in Figure 2. After adjusting for confounders, the odds of MAFLD exhibited a nonlinear decrease with increasing serum levels of total 25(OH)D 25(OH)D_3_, and epi-25(OH)D_3_ [total 25(OH)D, 25(OH)D_3_, and epi-25(OH)D_3_: all *p*-nonlinear < 0.001].

### 3.3. Subgroup Analyses of the Relationship Between 25(OH)D and MAFLD

**Figure 3:**
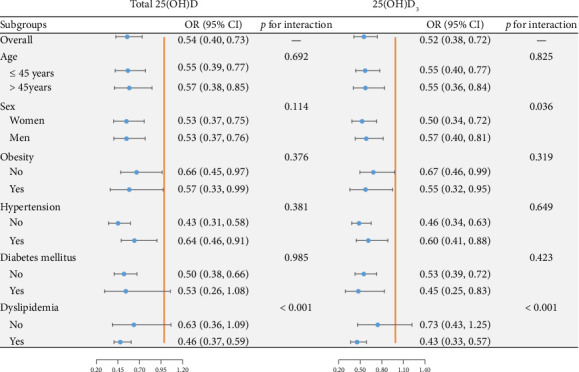
Subgroup analyses of total 25(OH)D and 25(OH)D_3_ with MAFLD. Both metabolites showed overall inverse associations with MAFLD across most subgroups. A significant sex-specific interaction was observed for 25(OH)D_3_, with stronger protective effects in women than in men (*p* for interaction = 0.036). In addition, dyslipidemia modified the associations for both total 25(OH)D and 25(OH)D_3_, with inverse relationships confined to participants with dyslipidemia (both *p* for interaction < 0.001).

Figure 3 shows the associations of 25(OH)D with MAFLD within various subgroups. After adjusting for age, sex, race, education levels, physical activity, family income–poverty ratio, smoking and drinking conditions, and the sampling season, the association of total 25(OH)D and 25(OH)D_3_ with MAFLD was stable in the age (< 45, ≥ 45), obesity (no, yes), hypertension (no, yes), and DM (no, yes) subgroups (all *p* for interaction > 0.05). Tests for interaction demonstrated that associations of total 25(OH)D and 25(OH)D_3_ with MAFLD were statistically different in participants with and without dyslipidemia. After stratifying participants by lipid status, only dyslipidemic participants showed negative associations of total 25(OH)D and 25(OH)D_3_ with MAFLD (all *p* for interaction < 0.001). In addition, a significant interaction by sex was observed for 25(OH)D_3_ (*p* for interaction = 0.036), with stronger inverse associations in women (OR = 0.50 [0.34–0.72]) than in men [OR = 0.57 [0.40–0.81]), whereas no such difference was found for total 25(OH)D.

## 4. Discussion

In this nationally representative sample from NHANES 2017–2018, we observed that low serum levels of total 25(OH)D, 25(OH)D_3_, and epi-25(OH)D_3_ were significantly associated with a higher prevalence of MAFLD. Subgroup analyses further revealed that these inverse associations were particularly evident among participants with dyslipidemia, whereas no significant relationship was observed in those without dyslipidemia. In addition, a sex-specific interaction was identified for 25(OH)D_3_, with stronger protective associations in women than in men, while no such difference was found for total 25(OH)D. It is noteworthy that these associations only emerged after multivariable adjustment, indicating that negative confounding by demographic and lifestyle factors may have obscured the true relationships in the unadjusted analyses.

Previous research has focused on the association between vitamin D and NAFLD, an earlier concept preceding the current definition of MAFLD, with most studies reporting an inverse relationship between NAFLD prevalence and serum 25(OH)D concentrations. In the Chinese population, low vitamin D levels have been related to increased susceptibility to NAFLD [19–21]. A cross-sectional study conducted at the Seoul National University Hospital found that vitamin D levels were adversely and dose-dependently correlated with NAFLD [22]. Moreover, results from NHANES 2017–2018 showed that serum 25(OH)D levels were negatively correlated with the likelihood of fibrosis and NAFLD [9]. According to a systematic review, multiple genetic polymorphisms related to vitamin D were linked to the presence of NAFLD [23]. Furthermore, in children with NAFLD, vitamin D was found to be inversely correlated with nonalcoholic steatohepatitis (NASH) and fibrosis [24]. Besides, a retrospective cohort study including 139,599 Korean adults showed that NAFLD incidence decreased over the survey period due to an increase in 25(OH)D, and NAFLD resolution was aided over time by adequate 25(OH)D [10].

There was disagreement on the relationship between serum 25(OH)D and the severity of NAFLD. On the one hand, in the general population from Florida, vitamin D concentrations were not related to liver fat accumulation or the severity of NASH [25]. A systematic review and meta-analysis involving 974 NAFLD patients from 6 studies also demonstrated no connection between serum 25(OH)D and the histological severity of NAFLD [26]. On the other hand, in biopsy-proven NAFLD patients, serum 25(OH)D deficiency was closely related to the degree of hepatic steatosis, necroinflammation, and fibrosis histologically [27]. Data from NHANES III showed that, in participants with NAFLD, vitamin D deficiency was related to liver steatosis and fibrosis severity [28]. Moreover, low plasma vitamin D levels were associated with the severity of hepatocyte ballooning and liver fibrosis, NAFLD activity score, and body fat mass [29, 30].

Few studies investigated the association between 25(OH)D and MAFLD. The Korea NHANES 2010–2011 proved no statistical association between MAFLD and vitamin D levels [31]. A population-based study recruiting 83,625 participants from Wenzhou, China, showed the nonlinear relationship between vitamin D and the presence of MAFLD; vitamin D was inversely correlated to MAFLD when vitamin D levels were ≥ 44.6 nmol/L; below this level, vitamin D might accelerate the progression of MAFLD [32]. However, no previous research has specifically examined the role of epi-25(OH)D_3_ in MAFLD. Epi-25(OH)D_3_ is a C-3 epimer of 25(OH)D_3_ generated during vitamin D metabolism. It also contributes to the circulating vitamin D pool but may not fully replicate the physiological actions of 25(OH)D_3_. Notably, higher proportions of epi-25(OH)D_3_ have been observed in newborns and pregnant women, suggesting potential developmental and metabolic implications [33]. Importantly, our study based on NHANES 2017–2018 found that low levels of total 25(OH)D, 25(OH)D_3_, and epi-25(OH)D_3_ were significantly associated with a higher prevalence of MAFLD. These associations were particularly evident in participants with dyslipidemia, and a sex-specific interaction was identified for 25(OH)D_3_, with stronger protective effects observed in women than in men.

The stronger inverse associations of vitamin D with MAFLD observed in dyslipidemic participants may be explained by the close interplay between vitamin D metabolism and lipid homeostasis. Vitamin D deficiency has been linked to adverse lipid profiles, including elevated triglycerides and reduced HDL-C, which are well-established risk factors for hepatic steatosis [34]. Moreover, vitamin D regulates hepatic lipid metabolism via modulation of sterol regulatory element-binding protein-1c and peroxisome proliferator-activated receptor (PPAR) pathways, both central to lipogenesis and fatty acid oxidation [35–37]. Dyslipidemia may therefore amplify the impact of vitamin D deficiency on hepatic fat accumulation and metabolic dysfunction. In addition, the observed sex-specific interactions could reflect the influence of sex hormones on vitamin D metabolism and liver disease risk. Estrogen has been reported to enhance vitamin D receptor activity and modulate lipid metabolism [38], which may strengthen the protective role of vitamin D in women. Conversely, men are more prone to visceral adiposity and insulin resistance [39]. Potentially attenuating the beneficial effects of vitamin D. These biological mechanisms together may account for the stronger inverse associations of 25(OH)D_3_ with MAFLD in dyslipidemic participants and in women compared with men.

Given that the majority of studies have revealed adverse effects of low vitamin D levels on NAFLD and metabolism, animal experiments have been conducted to delve into the mechanisms underpinning the connections between vitamin D and metabolic disorders. In obese Sprague–Dawley rats, vitamin D deficiency worsened NAFLD by triggering Toll-like receptors and upregulating genes of liver inflammation and oxidative stress [40]. Vitamin D alleviated liver injury in high-fat fed rats by inhibiting pyroptosis, inducing alterations in gut microbiota and metabolism [41, 42], and activating the PPARα pathway [35]. Furthermore, the vitamin D analog calcipotriol was proven to inhibit liver inflammation and hepatic steatosis via the activation of vitamin D receptors [43]. Vitamin D treatment suppressed the p53 pathway, thereby slowing the progression of NAFLD [44].

The effects of vitamin D administration on liver injury and metabolism are also contradictory. In patients with type 2 DM, high doses of vitamin D treatment (cholecalciferol 2000 IU/day) for 24 weeks did not lead to reduction in liver steatosis or improvements in metabolic abnormalities [45]. Likewise, vitamin D supplementation had no impact on serum hepatic enzymes in overweight or obese adults [46]. Nonetheless, in a randomized, double-blind trial, supplementation of vitamin D (1000 IU/day) (*n* = 201) for 48 weeks decreased liver steatosis and fibrosis [47]. A 12-week treatment of vitamin D (3200 IU) resulted in reduced ALT levels in women with polycystic ovary syndrome [48]. Moreover, a systematic review and meta-analysis, encompassing 16 randomized controlled trials, conclusively illustrated that vitamin D exerted a significant influence on weight, BMI, HDL-C, FPG, ALT, and HOMA-IR [49]. Potentially, factors such as sex differences, baseline vitamin D levels, and genetic polymorphisms may play a role in modulating the effects of vitamin D supplementation, which should be considered in future clinical trials.

This study had several limitations. First, due to the cross-sectional design, a causal relationship between serum 25(OH)D and MAFLD cannot be established, and the possibility of reverse or bidirectional causation cannot be excluded. Second, serum 25(OH)D was measured only once, which may not fully capture long-term vitamin D status, although a single measurement has been considered reliable in epidemiological settings [50]. Third, information on recent vitamin D supplementation was not available in the NHANES dataset, and we were therefore unable to exclude such participants, which may have influenced the results. Finally, despite extensive adjustment for confounders, residual confounding may remain, particularly since parathyroid hormone was not measured in NHANES 2017–2018.

## 5. Conclusions

In conclusion, lower serum concentrations of total 25(OH)D, 25(OH)D_3_, and epi-25(OH)D_3_ were independently associated with an increased prevalence of MAFLD, with stronger associations observed in participants with dyslipidemia. In addition, 25(OH)D_3_ showed a sex-specific interaction, demonstrating greater protective effects in women than in men. As this was a cross-sectional study, causal inference is limited, and reverse or bidirectional associations remain possible; therefore, further longitudinal and mechanistic studies are needed to clarify the temporal relationship and underlying pathways between vitamin D status and MAFLD.

## Figures and Tables

**Table 1:** Characteristics of participants categorized by the presence of MAFLD.

Variables	Total	Non-MAFLD	MAFLD	*p*
Participants (%)	4065 (100)	1958 (45.09)	2647 (54.91)	—
Age, years	48.01 ± 0.67	44.20 ± 0.73	51.13 ± 0.67	< 0.001
Sex (%)				< 0.001
Women	2352 (51.20)	1103 (57.69)	1249 (45.86)	
Men	2253 (48.80)	855 (42.31)	1398 (54.14)	
Ethnicity (%)				< 0.001
Non-Hispanic White	1587 (62.70)	678 (64.12)	909 (61.54)	
Non-Hispanic Black	1044 (10.82)	517 (12.51)	527 (9.44)	
Mexican American	638 (9.01)	194 (6.38)	444 (11.18)	
Other	1336 (17.46)	569 (16.99)	767 (17.84)	
Education (%)				0.004
College graduate or above	1110 (31.13)	517 (35.02)	593 (27.98)	
Some college or aa degree	1490 (30.55)	618 (28.68)	872 (32.13)	
High school graduate	1096 (27.31)	461 (26.31)	635 (28.16)	
Less than 12th	901 (10.94)	360 (9.98)	541 (11.73)	
Smoking (%)				0.231
No	3774 (82.69)	1559 (81.74)	2215 (83.48)	
Yes	831 (17.31)	399 (18.26)	432 (16.52)	
Drinking (%)				0.125
No	1335 (22.71)	537 (20.91)	798 (24.19)	
Yes	3006 (73.21)	1302 (75.46)	1704 (71.35)	
Missing	264 (4.08)	119 (3.62)	145 (4.45)	
Physical activity (%)				< 0.001
Low	563 (11.13)	215 (7.54)	348 (14.08)	
Moderate	2064 (49.89)	934 (54.34)	1130 (46.23)	
High	821 (18.87)	372 (20.64)	449 (17.41)	
Missing	1157 (20.11)	437 (17.47)	720 (22.28)	
Family income–poverty ratio (%)				0.511
≤ 1	747 (11.30)	345 (11.86)	402 (10.84)	
1–3	1770 (31.56)	721 (30.43)	1049 (32.48)	
> 3	1507 (46.99)	655 (48.33)	852 (45.89)	
Missing	581 (10.15)	237 (9.37)	344 (10.79)	
Hypertension (%)	2124 (39.20)	664 (24.90)	1460 (50.95)	< 0.001
Diabetes mellitus (%)	795 (12.33)	152 (3.84)	643 (19.31)	< 0.001
Dyslipidemia (%)	3199 (67.12)	1085 (53.51)	2114 (78.30)	< 0.001
BMI (kg/m^2^)	29.76 ± 0.26	25.72 ± 0.27	33.04 ± 0.33	< 0.001
Waist circumference (cm)	100.68 ± 0.69	89.81 ± 0.66	109.61 ± 0.82	< 0.001
Blood pressure (mmHg)				
Systolic	123.46 ± 0.55	119.05 ± 0.61	127.10 ± 0.57	< 0.001
Diastolic	72.91 ± 0.54	71.27 ± 0.65	74.27 ± 0.58	< 0.001
TC (mmol/L)	4.90 ± 0.04	4.80 ± 0.04	4.98 ± 0.05	0.002
TG (mmol/L)	1.27 ± 0.04	0.94 ± 0.03	1.55 ± 0.07	< 0.001
HDL-C (mmol/L)	1.38 ± 0.01	1.52 ± 0.01	1.27 ± 0.01	< 0.001
LDL-C (mmol/L)	2.88 ± 0.04	2.82 ± 0.03	2.93 ± 0.06	0.080
FPG (mmol/L)	6.11 ± 0.06	5.58 ± 0.03	6.55 ± 0.10	< 0.001
Insulin (mU/L)	13.14 ± 0.43	8.20 ± 0.34	17.24 ± 0.60	< 0.001
HOMA-IR	3.88 ± 0.16	2.10 ± 0.10	5.35 ± 0.23	< 0.001
HbA1c (%)	5.68 ± 0.02	5.40 ± 0.01	5.91 ± 0.03	< 0.001
hs-CRP (mg/L)	3.79 ± 0.16	2.60 ± 0.20	4.77 ± 0.20	< 0.001
ALT (U/L)	23.13 ± 0.40	19.34 ± 0.60	26.24 ± 0.62	< 0.001
AST (U/L)	22.17 ± 0.26	21.30 ± 0.52	22.88 ± 0.43	0.066
Sampling season (%)				0.761
May 1 through October 31	2359 (54.03)	988 (54.44)	1371 (53.69)	
November 1 through April 30	2246 (45.97)	970 (45.56)	1276 (46.31)	
Total 25(OH)D (nmol/L)	73.06 ± 1.60	74.49 ± 1.25	71.88 ± 2.03	0.051
25(OH)D_2_ (%)				0.480
≤ 1.45 nmol/L	3668 (80.50)	1565 (79.52)	2103 (81.30)	
> 1.45 nmol/L	937 (19.50)	393 (20.48)	544 (18.70)	
25(OH)D_3_ (nmol/L)	69.48 ± 1.59	71.58 ± 1.16	67.77 ± 2.16	0.023
epi-25(OH)D_3_ (nmol/L)	4.59 ± 0.17	4.60 ± 0.11	4.58 ± 0.23	0.902
Median CAP (dB/m)	264.49 ± 1.76	209.46 ± 1.05	309.67 ± 1.63	< 0.001

*Note:* MAFLD, metabolic dysfunction-associated fatty liver disease; HbA1c, glycosylated hemoglobin; ALT, alanine aminotransferase; AST, aspartate aminotransferase; 25(OH)D, 25-hydroxyvitamin D; 25(OH)D_2_, 25-hydroxyvitamin D_2_; 25(OH)D_3_, 25-hydroxyvitamin D_3_; TG, triglycerides.

Abbreviations: BMI, body mass index; CAP, controlled attenuated parameter; FPG, fasting plasma glucose; HDL-C, high-density lipoprotein cholesterol; HOMA-IR, homeostasis model assessment of insulin resistance; hs-CRP, hypersensitive C-reactive protein; LDL-C, low-density lipoprotein cholesterol; TC, total cholesterol.

**Table 2:** Odds ratios of MAFLD by different status of 25(OH)D.

	Cases (%)	Model 1		Model 2		Model 3	
		OR (95% CI)	*p* value	OR (95% CI)	*p* value	OR (95% CI)	*p* value
Total 25(OH)D (nmol/L)							
Q1 (9.96, 46.80)	663 (58.37)	Reference	—	Reference	—	Reference	—
Q2 (46.80, 64.80)	679 (58.65)	1.01 (0.76, 1.34)	0.931	0.93 (0.69, 1.25)	0.611	0.89 (0.66, 1.20)	0.433
Q3 (64.80, 85.20)	672 (54.21)	0.84 (0.61, 1.17)	0.282	0.70 (0.49, 0.99)	0.047	0.68 (0.48, 0.98)	0.038
Q4 (85.20, 422.00)	633 (50.38)	0.72 (0.51, 1.03)	0.067	0.45 (0.29, 0.68)	0.002	0.45 (0.29, 0.68)	< 0.001
*p* for trend			0.032		< 0.001		< 0.001
As a continuous variable^†^	2647 (54.91)	0.79 (0.63, 0.99)	0.045	0.54 (0.41, 0.72)	< 0.001	0.54 (0.40, 0.73)	< 0.001
25(OH)D_3_ (nmol/L)							
Q1 (6.20, 42.10)	697 (62.12)	Reference	—	Reference	—	Reference	—
Q2 (42.10, 60.80)	657 (56.86)	0.80 (0.60, 1.07)	0.124	0.80 (0.60, 1.07)	0.124	0.70 (0.51, 0.95)	0.025
Q3 (60.80, 81.30)	655 (54.04)	0.72 (0.51, 1.00)	0.053	0.72 (0.51, 1.00)	0.053	0.58 (0.41, 0.84)	0.007
Q4 (81.30, 421.00)	638 (49.92)	0.61 (0.44, 0.85)	0.006	0.61 (0.44, 0.85)	0.006	0.39 (0.25, 0.59)	< 0.001
*p* for trend			0.009		0.009		< 0.001
As a continuous variable^†^	2647 (54.91)	0.73 (0.58, 0.93)	0.014	0.54 (0.40, 0.72)	< 0.001	0.52 (0.38, 0.72)	< 0.001
epi-25(OH)D_3_ (nmol/L)							
Q1 (1.16, 2.24)	659 (56.35)	Reference	—	Reference	—	Reference	—
Q2 (2.24, 3.38)	666 (58.43)	1.09 (0.92, 1.30)	0.306	1.03 (0.86, 1.23)	0.754	1.01 (0.85, 1.21)	0.861
Q3 (3.38, 4.99)	638 (51.78)	0.83 (0.62, 1.11)	0.187	0.69 (0.50, 0.96)	0.031	0.70 (0.51, 0.97)	0.036
Q4 (4.99, 37.70)	684 (54.44)	0.93 (0.68, 1.26)	0.593	0.61 (0.41, 0.91)	0.019	0.63 (0.42, 0.95)	0.029
*p* for trend			0.357		0.012		0.020
As a continuous variable^†^	2647 (54.91)	0.96 (0.79, 1.16)	0.651	0.74 (0.58, 0.94)	0.019	0.77 (0.60, 0.99)	0.041

*Note:* Model 1: unadjusted. Model 2: adjusted for age and gender. Model 3: adjusted for age, gender, race, education levels, family income–poverty ratio, physical activity levels, smoking and drinking conditions, and the sampling season. MAFLD, metabolic dysfunction-associated fatty liver disease; 25(OH)D, 25-hydroxyvitamin D; 25(OH)D_3_, 25-hydroxyvitamin D3.

Abbreviations: CI, confidence intervals; OR, odds ratio.

^†^Log-transformed with base natural constant.

## Data Availability

The data that support the findings of this study are openly available online (https://wwwn.cdc.gov/nchs/nhanes/default.aspx).

## Nomenclature

25(OH)D: 25-Hydroxyvitamin D
CAP: Controlled attenuated parameter
CI: Confidence interval
DM: Diabetes mellitus
FPG: Fasting plasma glucose
HbA1c: Glycosylated hemoglobin
HDL-C: High-density lipoprotein cholesterol
HOMA-IR: Homeostasis model assessment of insulin resistance
hs-CRP: Hypersensitive C reactive protein
LDL-C: Low-density lipoprotein cholesterol
MAFLD: Metabolic dysfunction-associated fatty liver disease
METs: Metabolic equivalents
NAFLD: Non-alcoholic fatty liver disease
NHANES: National Health and Nutrition Examination Survey
ORs: Odds ratios
SE: Standard error
TC: Total cholesterol
TG: Triglyceride

## Supporting Information

Supporting Table 1 Odds ratios of MAFLD by different status of 25(OH)D.
