# Supplementary material for: Association of 25-Hydroxyvitamin D With Metabolic Dysfunction-Associated Fatty Liver Disease: Results From NHANES 2017–2018
Source: Int J Endocrinol. 2025 Sep 23;2025:1368301. doi: 10.1155/ije/1368301 (PMC12483731; doi:10.1155/ije/1368301)
Supplement: Supporting Information — Additional supporting information can be found online in the Supporting Information section. [file 1368301.f1.docx]

Supplemental Table 1 Odds ratios of MAFLD by different status of 25(OH)D.

|  | Cases (%) | Model 1 | |  | Model 2 | |  | Model 3 | |
| --- | --- | --- | --- | --- | --- | --- | --- | --- | --- |
|  |  | OR (95% CI) | P value |  | OR (95% CI) | P value |  | OR (95% CI) | P value |
| Deficiency (9.96,30.00) | 218(58.63) | 1.18(0.88,1.59) | 0.248 |  | 1.53(1.12,2.09) | 0.013 |  | 1.63(1.19,2.23) | 0.005 |
| Inadequate (30.00,50.00) | 548(57.12) | 1.11(0.82,1.51) | 0.471 |  | 1.34(0.97,1.85) | 0.071 |  | 1.27(0.95,1.71) | 0.104 |
| Adequate (50.00,125.00) | 1764(54.53) | Reference | - |  | Reference | - |  | Reference | - |
| Excess (125.00,422.00) | 117(50.14) | 0.84(0.57,1.24) | 0.349 |  | 0.67(0.45,0.99) | 0.045 |  | 0.69(0.47,1.02) | 0.060 |

MAFLD, Metabolic dysfunction-associated fatty liver disease; 25(OH)D, 25-hydroxyvitamin D; OR, odds ratio; CI, confidence intervals.

Model 1: unadjusted.

Model 2: adjusted for age and gender.

Model 3: adjusted for age, gender, race, education levels, family income-poverty ratio, physical activity levels, smoking and drinking conditions, and the sampling season.
